# Evolving role of proton therapy in contemporary oncology

**DOI:** 10.1002/cncr.70622

**Published:** 2026-10-13

**Authors:** Emily S. Lebow, Helen A. Shih

**Affiliations:** ^1^ Department of Radiation Oncology University of Pennsylvania Philadelphia Pennsylvania USA; ^2^ Department of Radiation Oncology Mass General Brigham Cancer Institute Massachusetts General Hospital Boston Massachusetts USA

**Keywords:** comparative effectiveness, patient selection, proton beam therapy, proton therapy, radiation oncology, randomized clinical trials, treatment‐related toxicity

## Abstract

Proton therapy offers a distinct physical advantage over photon radiotherapy through the Bragg peak dose concentration in target with both lower integral dose and finite path length, and sharper penumbra as compared to photons, collectively reducing radiation exposure to surrounding normal tissues and potentially improving the therapeutic ratio. However, this advantage is tempered by uncertainties, including range uncertainty and variable relative biological effectiveness. In this review, the authors summarize contemporary evidence supporting proton therapy across pediatric, central nervous system (CNS), head and neck, thoracic, breast, gastrointestinal, genitourinary, and hematologic malignancies, as well as reirradiation. The strength of evidence varies substantially by disease site. Proton therapy is well established in pediatric cancers and selected CNS tumors, where minimizing integral dose and late toxicity is especially important. Randomized trials have demonstrated clinically meaningful benefits in selected settings, including improved disease control and survival with proton craniospinal irradiation for leptomeningeal metastases and reduced toxicity with preserved disease control and survival benefit in oropharyngeal cancer. Conversely, randomized data in localized prostate cancer have not shown improved patient reported outcomes compared with intensity modulated photon therapy. Ongoing phase 3 trials across multiple disease sites are expected to further define the role of protons by clarifying comparative effectiveness, toxicity reduction, and cost‐effectiveness relative to advanced photon techniques. Future progress will depend on improved patient selection, robust treatment planning, and emerging technologies including adaptive proton therapy, proton arc therapy, and FLASH radiotherapy.

## Abstract 2

Proton therapy offers a distinct radiation dose distribution characterized by the absence of exit dose and the potential to reduce radiation exposure to surrounding normal tissues as compared to photon therapy. In this review, the authors synthesize the current evidence across major disease sites, highlight areas of demonstrated and potential benefit, and discuss ongoing trials that are likely to further clarify the role of proton therapy in modern oncology.

## INTRODUCTION

Proton therapy is distinguished by its physical dose distribution, characterized by the Bragg peak, which allows radiation dose to be deposited in a more confined region with reduced exposure to surrounding normal tissues compared with conventional photon therapy (Figure 1). This feature has the potential to improve the therapeutic ratio, particularly for tumors located near critical radiation sensitive tissues.^2^ However, proton therapy subject to greater uncertainty. This includes particle range and variable energy transfer at the distal edge of the beam, necessitating robust planning, optimization, and image guidance for proton therapy planning.^3^

**FIGURE 1:**
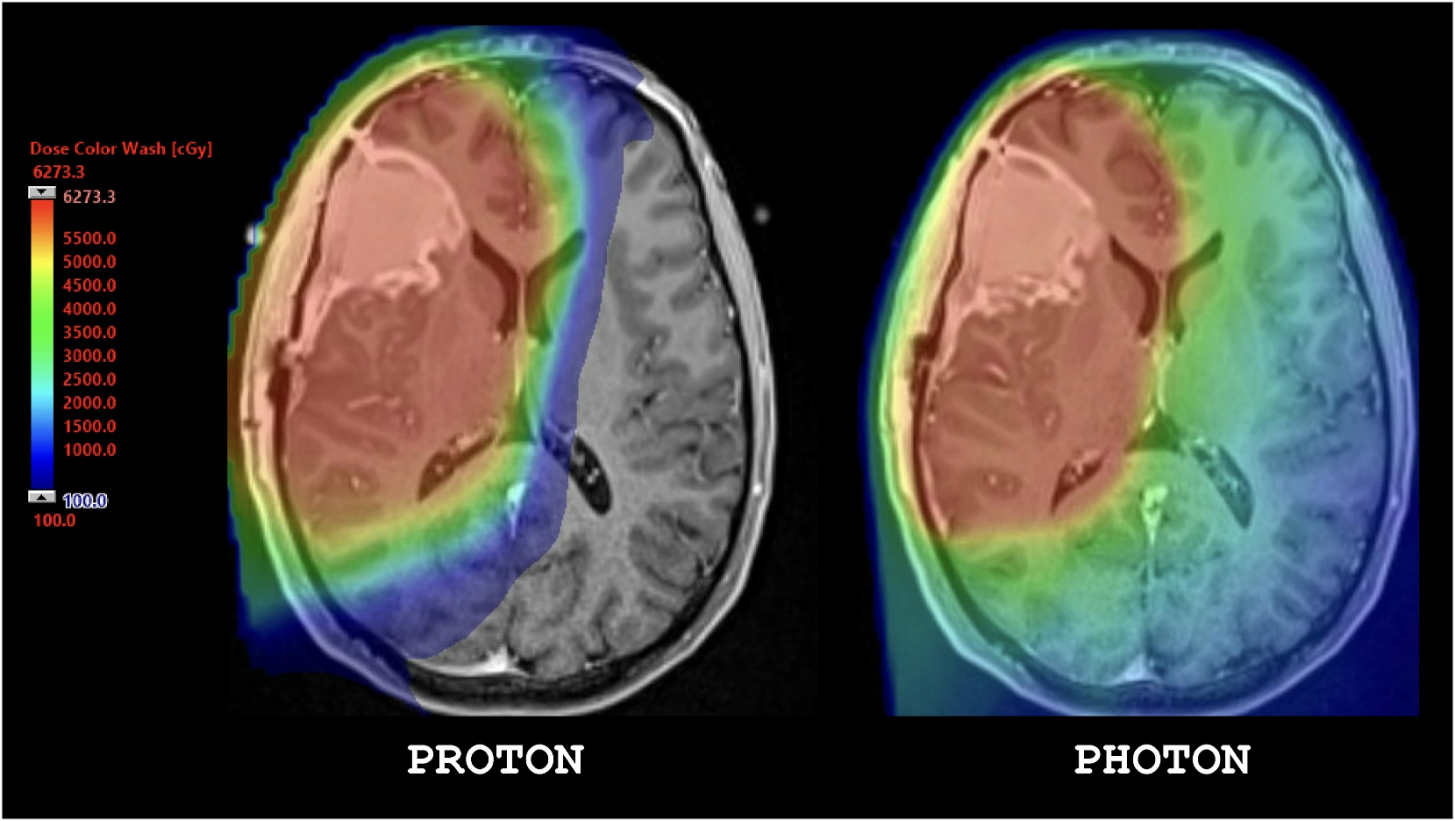
Representative axial dose distribution comparing proton (*left*) versus photon (*right*) radiotherapy plans for a patient with a resected WHO grade 3 IDH mutant astrocytoma. Both plans achieve comparable target coverage. However, the proton plan demonstrates improved conformality with reduced low‐ and intermediate‐dose spill to the contralateral hemisphere and surrounding normal brain. This reduction in integral dose highlights the potential advantage of proton therapy in minimizing irradiation of uninvolved brain tissue. A randomized comparison of proton versus photon radiation for patients with WHO grade 2 or 3 IDH mutant glioma has completed accrual as part of NRG BN005 with a primary end point of cognitive toxicity.^1^

The clinical adoption of proton therapy has been accompanied by ongoing debate. Higher treatment costs^4^, ^5^, ^6^ and inconsistent evidence demonstrating improvements in patient outcomes compared with photon radiotherapy^7^, ^8^ have contributed to uncertainty regarding its optimal role across disease sites. At the same time, advances in photon therapy, including intensity modulated radiotherapy (IMRT), and specifically volumetric modulated arc therapy (VMAT), image guidance, and adaptive treatment strategies, have enabled increasingly conformal dose delivery with improved normal tissue sparing, delivered with higher efficiency.^9^ As a result, the gap between photon and proton techniques has narrowed in many clinical settings.

The central question surrounding proton therapy is whether the physical properties of proton therapy translate into clinical benefits. These potential benefits may include reductions in toxicity, improvements in quality of life, greater tolerance of multimodality therapy, and improved survival. Recent prospective and randomized studies have begun to provide more direct evidence regarding the clinical impact of proton therapy, although the strength of evidence varies across disease sites (Table 1).

**TABLE 1:** Evidence evaluating proton therapy across major disease sites.

Disease site	Retrospective^a^	Prospective, single arm	Randomized phase 3 trials initiated	Randomized phase 3 trials reported	Key references
Pediatric	✓	✓			^10^, ^11^, ^12^
Central nervous system	✓	✓	✓	✓	^13^, ^14^, ^15^, ^16^, ^17^
Head and neck	✓	✓	✓	✓	^7^, ^8^
Lung	✓	✓	✓		^18^, ^19^
Breast	✓	✓	✓		^20^, ^21^
Esophageal	✓	✓	✓		^22^
Hepatobiliary	✓	✓	✓		^23^
Genitourinary	✓	✓	✓	✓	^24^
Hematologic	✓	✓			^25^
Reirradiation	✓	✓			^26^, ^27^

*Note*: Early studies primarily demonstrated dosimetric advantages relative to photon therapy. Subsequent retrospective analyses and prospective cohort studies have evaluated clinical outcomes and toxicity. More recently, randomized trials in select disease sites are providing higher level evidence regarding the value of proton therapy.

^a^ Retrospective studies include dosimetric analyses comparing dose distribution of proton versus photon therapy as well as clinical reports.

In this review, we summarize the current evidence supporting the use of proton therapy across major disease sites, including pediatric malignancies, central nervous system (CNS) tumors, head and neck cancers, thoracic malignancies, breast cancer, gastrointestinal, genitourinary, and hematologic cancers (Table 2). We further discuss ongoing studies that are likely to further define the role of proton therapy in modern oncology.

**TABLE 2:** Select key studies of proton versus photon therapy across major disease sites.

Disease site	Trial	Phase	Population	Comparator	Primary end point(s)	Status/key findings
CNS	NRG BN001^16^	2	CNS WHO grade 4 IDH‐wildtype glioblastoma	Dose intensified vs. conventional radiation	OS	Dose intensified proton cohort met study criteria for improved survival and future study in phase 3 setting
CNS	NRG BN005^1^	2	CNS WHO grade 2 or 3, IDH‐mutant, astrocytoma or oligodendroglioma	Proton vs. photon	Change in cognition	Accrual completed
CNS	NRG BN014 (RADIATE‐LM)^28^	3	Leptomeningeal disease (breast or lung)	Proton craniospinal irradiation vs. involved field photon	OS	Open for accrual
Head and neck	Frank et al. (NCT01893307)^7^	3	Oropharyngeal cancer	Proton vs. photon	Noninferior PFS	Proton met criteria for noninferior PFS and associated with improved OS and reduced high grade toxicity
Head and neck	TORPEdO^8^	3	Oropharyngeal cancer	Proton vs. photon	Coprimary end points of 12‐month gastronomy tube dependence or severe (>20%) weight loss and composite patient reported QOL	No difference in functional or QOL outcomes No difference in PFS or OS
Thoracic	RTOG 1308 (NCT01993810)^29^	3	Stage II–III non–small cell lung cancer	Proton vs. photon	OS	Accrual completed
Thoracic	NRG GI006 (NCT03801876)^30^	3	Esophageal cancer	Proton vs. photon	Coprimary end points of OS and reduced grade 3 or greater cardiopulmonary toxicity	Open for accrual
Thoracic	PROTECT (NCT050555648)^31^	3	Esophageal cancer	Proton vs. photon	Incidence of pulmonary complications through 90 days after surgery	Open for accrual
Breast	RADCOMP (NCT02603341)^21^	3	Locally advanced breast cancer	Proton vs. photon	10‐year cardiovascular events	Accrual completed
Gastrointestinal	NRG GI003 (NCT03186898)^32^	3	Unresectable or locally recurrent hepatocellular carcinoma	Proton vs. photon	OS	Open for accrual
Prostate	PARTIQoL Trial (NCT01617161)^24^	3	Localized prostate cancer	Proton vs. photon	Change in baseline bowel quality of life at 24 months	No difference in bowel function at 24 months No difference in urinary or sexual function or PFS

Abbreviations: CNS, central nervous system; OS, overall survival; PFS, progression‐free survival; QOL, quality of life; WHO, World Health Organization.

### Pediatric malignancies

The most widely accepted indication for proton therapy is in pediatric malignancies, where the goal is to minimize long‐term treatment‐related toxicity in patients with potentially long life expectancy.^33^ Although randomized trials are lacking, a large body of dosimetric, prospective, and retrospective data consistently demonstrates that proton therapy reduces radiation exposure to normal tissues while maintaining tumor control outcomes comparable to photon‐based radiotherapy.^33^ It is important to note that based on the described physical principles of proton therapy, regardless of the form of advanced photon technology that proton therapy is compared to, proton therapy will virtually always have substantially less low dose radiation collateral exposure to nontarget normal tissues (Figure 1). Where very low dose radiation may be clinically insignificant in many adults, there is often a significant risk for long‐term negative sequelae in pediatric patients.

The strongest clinical evidence exists in pediatric CNS tumors, especially those requiring radiation of the entire neuraxis (craniospinal irradiation), where proton therapy significantly reduces dose to the heart, lungs, thyroid, and gastrointestinal organs. Clinical studies have shown lower rates of treatment‐related toxicities, including ototoxicity, endocrine dysfunction, and cognitive decline,^34^, ^35^, ^36^ while maintaining disease control comparable to photon‐based cooperative group trials.^10^, ^36^, ^37^, ^38^, ^39^, ^40^ Similar advantages have been reported in other intracranial tumors including ependymoma,^41^ germinoma,^42^, ^43^ craniopharyngioma,^11^ and low grade glioma,^44^, ^45^, ^46^ as well as in in extracranial sites such as rhabdomyosarcoma and Ewing sarcoma, where improved sparing of surrounding normal tissues may reduce long‐term morbidity.^47^, ^48^

Physical properties of proton therapy also require special consideration and robust planning at experienced proton centers. Proton dose distributions are sensitive to anatomic changes during treatment,^49^ and uncertainties related to proton range and variable relative biological effectiveness (RBE), particularly near the distal edge of the beam,^3^ require careful consideration when the target abuts critical structures. These challenges are particularly relevant among patients with craniopharyngioma, where fluctuations in cyst volume during the course of radiation raise concern for geographic target misses or overdosing critical structures such as the optic chiasm.^11^, ^49^ Consequently, careful magnetic resonance imaging surveillance throughout treatment, robust treatment planning, and adaptive replanning when indicated are essential components of proton therapy for these patients. Early reports of brainstem imaging changes and radiation injury following proton therapy also prompted concerns regarding end‐of‐range effects and elevated RBE and linear energy transfer (LET) near the distal edge of the beam.^50^, ^51^ Although subsequent clinical experience and improved treatment planning techniques and conservative dose constraints have mitigated many of these concerns,^52^, ^53^ these observations underscore the importance of robust planning when treating tumors adjacent to critical structures.

### CNS

Proton therapy has an important role in the management of brain malignancies, where the proximity of tumors to critical neurologic structures makes normal tissue sparing particularly relevant. A substantial body of evidence has established the relationship between radiation dose to critical brain structures and subsequent neurocognitive, endocrine, and auditory toxicity. Prospective and randomized data have demonstrated that reducing radiation dose to the hippocampi and temporal lobes preserves cognitive function following cranial irradiation.^54^, ^55^, ^56^ There are also well established radiation dose–response relationships for endocrine dysfunction following irradiation of the hypothalamic–pituitary axis^57^, ^58^ and sensorineural hearing loss with treatment of the cochlea, providing an additional rationale for minimizing dose to the auditory apparatus when feasible.^59^

Among adult patients, proton therapy is considered a standard approach for skull base chordomas and chondrosarcomas, where durable local control requires high radiation doses in close proximity to the brainstem, optic pathways, and temporal lobes.^60^ The strongest prospective evidence comes from PROG 85‐26, a randomized dose escalation study in patients with skull base and cervical chordomas and chondrosarcomas, which demonstrated the feasibility, safety, and efficacy of high dose proton therapy with a median follow‐up of over 29 years.^13^, ^61^ These data have established proton therapy as a preferred modality in this setting. Proton therapy is also frequently used in the management of meningioma and pituitary adenoma,^62^, ^63^ where long‐term disease control is expected among most patients and minimizing late toxicity is a central treatment goal despite the lack of randomized data.

The role of proton therapy continues to be investigated in diffuse gliomas, particularly among patients with grade 2 or grade 3 IDH‐mutant diffuse gliomas (Figure 1). These patients often experience prolonged survival and are therefore at increased risk for late toxicity. A prospective phase 2 study of 60 patients with low grade gliomas treated with proton therapy with median follow‐up of 7 years showed favorable tumor control and toxicity outcomes, with data suggesting preservation of neurocognitive function, neuro‐endocrine, and quality of life in the majority of patients.^14^, ^64^, ^65^ Additional retrospective studies suggest comparable or improved outcomes with proton therapy for this patient population.^66^, ^67^ In contrast, a recent retrospective multi‐institutional conference abstract reported inferior next intervention‐free survival, progression‐free survival (PFS), and overall survival (OS) patients with grade 3 IDH‐mutated gliomas treated with proton therapy compared with photon therapy.^68^ These findings have generated considerable interest but should be interpreted cautiously given the retrospective design, potential selection bias and unmeasured confounding, reliance on subgroup analyses, and the unexpectedly large magnitude of the reported survival differences, which lacks a clear biologic rationale. Consequently, these results should be considered hypothesis generating rather than practice changing. Several randomized trials are expected to better define the role of proton therapy in diffuse gliomas. A randomized phase 2 trial comparing proton versus photon therapy with a primary end point of cognitive preservation (NRG BN005; NCT03180502) has completed accrual and is expected to provide important data.^1^ Additional randomized data are expected as well for a focus on quality of life and functional outcomes.^69^, ^70^, ^71^

In grade 4 IDH–wild‐type glioblastoma, the role of proton therapy continues to be investigated. A randomized phase 2 trial demonstrated reduced patient reported fatigue with proton therapy compared to photon therapy, but no differences in cognitive or survival outcomes.^15^ Additional prospective data come from NRG BN001. BN001 was designed as a signal seeking, randomized phase 2 trial of dose intensified (75 Gy) versus standard dose (60 Gy) radiation among patients with glioblastoma.^16^ An analysis of the dose intensified proton cohort demonstrated a 6.8% improvement in 2‐year OS without an increase in high grade toxicity. This encouraging survival benefit is planned for further evaluation in a phase 3 study. Additional randomized data are also expected.^72^

Although proton therapy is generally associated with reduced integral dose and a favorable normal tissue dosimetric profile, its unique physical and biological characteristics have prompted investigation into potential proton specific toxicities within the CNS. Although clinical evidence remains inconclusive, uncertainty surrounding RBE and elevated linear energy transfer LET near the distal edge of the proton beam has been proposed as a mechanism contributing to radiation necrosis and pseudoprogression, particularly when critical structures are located adjacent to the beam end range.^47^, ^73^ This issue is also relevant for skull base chordomas and chondrosarcomas, where treatment places the distal edge of the beam near the brainstem or temporal lobes, and rare cases of brainstem injury or temporal lobe necrosis have been reported.^74^ Similarly, in primary brain tumors, several studies have described distinctive radiographic changes following proton therapy.^75^ As proton therapy continues to expand for CNS malignancies, further prospective studies integrating LET and variable RBE modeling will be essential to optimize treatment planning for patients treated with proton therapy.^3^

### Leptomeningeal disease

Proton therapy has also gained increasing attention for the treatment of leptomeningeal disease (LMD) and other indications requiring treatment of the entire neuraxis with craniospinal irradiation (CSI). In a phase 2 randomized trial, patients with leptomeningeal metastases from solid tumors were randomized to receive proton CSI versus involved field photon radiotherapy. Proton CSI significantly improved CNS PFS, overall PFS, and OS compared with photon involved field radiotherapy without a significant increase in severe treatment‐related toxicity. The survival benefit observed in this trial was attributed to the improved CNS disease control as there was a significant reduction in the proportion of patients who died with CNS progression among those who received proton CSI.^17^ The survival benefit of proton CSI is particularly notable in LMD that has historically been extremely difficult to treat due to limited effective systemic therapy options.^76^, ^77^ Given these encouraging results, proton CSI is increasingly being considered for selected patients with leptomeningeal metastases who have adequate performance status and controlled systemic disease. Proton CSI for LMD is currently undergoing evaluation in a randomized, phase 3 setting in NRG BN014 (RADIATE‐LM).^28^

### Head and neck

In head and neck cancer, the role of proton therapy has recently been informed by randomized phase 3 data. Two randomized phase 3 trials published in 2026 have evaluated proton therapy for head and neck cancer. A US‐based trial randomized 440 patients with locally advanced oropharyngeal cancer to proton versus photon (IMRT) with concurrent systemic therapy, using noninferior PFS as the primary end point. The trial showed that proton therapy had noninferior 5‐year PFS (81.3% vs. 76.2%; hazard ratio [HR], 0.88). In addition, an improvement in 5‐year OS was observed (90.9% vs. 81.0%; HR, 0.58), although this was a prespecified secondary end point. Furthermore, proton therapy was associated with lower rates of several clinically meaningful toxicities including lower rates of severe lymphopenia (76% vs. 89%), dysphagia (31% vs. 49%), xerostomia (33% vs. 45%), and significantly lower gastronomy tube dependence (26.8% vs. 40.2%, *p* = .018) compared with photon IMRT. Of particular clinical significance is the reduction in grade 3 or greater xerostomia because this toxicity is likely to be permanent and a detriment to quality of life. There was no difference in recurrence patterns between groups, including local, regional, and distant control. Notably, 23% of patients crossed over after randomization, largely due to issues with insurance coverage of proton therapy and systemic therapy was variable, but outcomes were similar in intention to treat and per protocol analyses. The authors hypothesized that improved survival may be related to reduction in treatment associated lymphopenia.^7^

The UK‐based Proton beam therapy for oropharyngeal cancer (TORPEdO) trial was also published in 2026 and offers a complementary perspective. This phase 3 study randomized 205 patients with locally advanced oropharyngeal cancer 2:1 to proton or photon therapy (IMRT) with uniform high dose cisplatin. The coprimary end points were functional outcomes including at 12 months gastrostomy dependence or severe weight loss greater than or equal to 20% and a composite patient reported quality of life score. There was no significant difference in either functional or quality of life outcomes. Both modalities showed excellent and comparable 24‐month locoregional control and OS. Proton therapy was associated with numerically lower rates of acute grade 3 or greater toxicity within the first 3 months of treatment including mucositis, dysphagia, and dysgeusia. These differences were not reflected in the primary end point because functional outcomes were assessed only at 12 months, when most acute treatment‐related toxicities had resolved. Late grade 3 toxicities were numerically higher among patients who received proton therapy despite lower incidence of early toxicity, though remained low overall, and was primarily driven by higher rates of late weight loss (14% vs. 5%).^8^ Nonetheless, the 12‐month composite quality of life score was not significantly different between patients who received proton or photon therapy.^8^

Taken together, the early results of these trials demonstrate that proton and photon therapy achieve comparable disease control and survival, whereas proton therapy is associated with a meaningful reduction in acute toxicity. This reduction is particularly relevant in the context of multimodality therapy with concurrent chemoradiation.^78^, ^79^ Although late toxicity differences remain less clearly defined, the overall pattern of preserved oncologic outcomes with improved early tolerability supports an enhanced therapeutic ratio with proton therapy, particularly for patients at higher risk of treatment toxicity. Long‐term data from both randomized trials with slightly different emphasis of survival toxicity will continue to help shape the role of radiotherapy for locally advanced oropharyngeal cancer. Additional randomized data are anticipated including the Danish Head and Neck Cancer Group 35 trial (NCT04607694) and the Swedish ARTSCAN V (NCT03829033).

### Thoracic

Proton therapy has the potential to improve the therapeutic ratio for thoracic malignancies by maintaining target coverage while reducing radiation dose to adjacent critical organs, including the heart, lungs, esophagus, and bone marrow. These dosimetric advantages may translate into lower cardiopulmonary toxicity, improved tolerance of multimodality therapy, and reduced lymphopenia. Radiation associated lymphopenia has become increasingly relevant given integration of immune checkpoint inhibition into standard of care treatment for curative intent thoracic malignancies.^80^ Retrospective evidence demonstrates that proton therapy reduces dose to circulating immune cells and the subsequent risk of radiation induced lymphopenia compared with photon therapy for patients with locally advanced lung cancer.^71^, ^81^, ^82^ Higher lymphocyte counts are associated with improved survival and disease control.^18^

The randomized evidence supporting proton therapy in thoracic malignancies is more limited and less definitive than in head and neck cancer. There are no completed phase 3 randomized studies to date. A randomized phase 2 study for locally advanced inoperable non–small cell lung cancer (NSCLC) comparing passive scatter proton therapy to photon IMRT showed no difference in the coprimary end points of rates of grade 3 or greater pneumonitis or local failure rates.^18^ The field has therefore largely been awaiting results from RTOG 1308, a definitive phase 3 trial that randomized patients with inoperable stage II–IIIB NSCLC to proton versus photon chemoradiation (70 Gy with concurrent platinum doublet chemotherapy) and is powered with a coprimary end point of OS and treatment‐related cardiac toxicity/lymphopenia.^19^ The biologic premise is that reducing heart and lung dose in light of prior data linking cardiac dose to survival in RTOG 0617 may translate into improved survival.^84^ The trial opened in 2014 and reached its accrual goal of 330 patients in 2023. The primary end point results are anticipated to be complete in December 2026, with secondary end point analyses expected the year afterward.^29^

The randomized evidence supporting proton therapy in esophageal cancer is somewhat more developed than in lung cancer, although phase 3 data remain limited. A randomized phase 2 trial of 145 patients comparing proton therapy with photon IMRT in patients with locally advanced esophageal cancer showed that proton therapy was associated with a significant reduction in a composite end point of total toxicity burden (TTB) that includes acute cardiopulmonary toxicity, postoperative complications, and treatment‐related morbidity. PFS and OS were similar.^78^ Two ongoing phase 3 studies are expected to define the role of proton therapy in esophageal cancer including: the US‐based NRG‐GI006 (NCT03801876)^30^ and European PROTECT trial (NCT05055648).^31^

### Breast

The evidence supporting proton therapy in breast cancer is evolving, with the strongest rationale centered on cardiac and lung dose reduction. Multiple dosimetric and early prospective studies have consistently demonstrated that proton therapy can substantially reduce mean heart dose, left anterior descending artery dose, and lung exposure compared with modern photon techniques.^85^ This is clinically relevant given well established data linking even low doses of cardiac radiation to adverse cardiac events.^86^, ^87^ Early prospective single arm studies showed low rates of acute toxicity and favorable early disease control, with emerging data suggesting reduced cardiac dose without compromising target coverage including for regional nodal irradiation.^88^, ^89^, ^90^ Proton therapy is also an important option for anatomically challenging scenarios including patients requiring regional nodal irradiation including the internal mammary nodes,^91^ treatment of patients with a pectus excavatum,^84^ and patients requiring synchronous bilateral breast irradiation.^90^

The Radiotherapy Comparative Effectiveness (RADCOMP) trial is a phase 3 randomized clinical trial comparing proton versus photon radiation therapy for patients with locally advanced breast cancer who require regional nodal irradiation. RADCOMP randomizes patients to receive standard of care photon radiotherapy (3D or IMRT) or to proton therapy. The primary end point is reduction in 10‐year major cardiac events (myocardial infarction, coronary revascularization, cardiovascular death, or major cardiac hospitalization). Key secondary end points include locoregional control, any recurrence, OS, and patient‐reported health‐related quality of life outcomes.^21^, ^92^ A challenge in interpreting the potential benefit of proton therapy in this setting is the long latency of radiation associated cardiac toxicity, requiring extended follow‐up.^85^ RADCOMP represents the only large phase 3 randomized effort in breast cancer, and its results will be pivotal in determining the role of proton therapy in this setting. RADCOMP is closed to accrual^93^ with primary end point results expected to be reported between 2028 and 2032.^94^

### Gastrointestinal and hepatobiliary

Proton therapy has emerged as a promising modality for the treatment of hepatocellular carcinoma (HCC), where preservation of uninvolved liver parenchyma is critical given the high prevalence of underlying cirrhosis and limited hepatic reserve. Furthermore, retrospective evidence suggests that higher doses of radiation are associated with improved local control and survival in selected patients.^95^, ^96^ Compared with photon radiotherapy, proton therapy can substantially reduce radiation dose to normal liver due to the absence of exit dose, which may lower the risk of radiation induced liver disease and enable safe dose escalation. Multiple prospective and retrospective series have reported excellent local control rates with proton therapy for HCC with favorable toxicity profiles even for tumors located near critical structures or in patients with borderline liver function.^97^, ^98^, ^99^ The randomized phase 3 NRG‐GI003 (NCT03186898) trial is currently comparing proton versus photon radiotherapy for patients with unresectable or locally recurrent HCC with OS as the primary end point.

### Genitourinary

In genitourinary malignancies, the largest body of clinical experience with proton therapy is in localized prostate cancer. Dosimetric studies have demonstrated reductions in integral dose and low to intermediate dose exposure to surrounding normal tissues, including the bladder, rectum, and pelvic bone marrow.^100^, ^101^, ^102^ These reductions are associated with lower rates of gastrointestinal and genitourinary toxicity in retrospective studies.^103^, ^104^, ^105^ Multiple cohort studies have reported safety and efficacy of proton therapy for localized prostate cancer with outcomes comparable to modern photon therapy.^106^, ^107^, ^108^, ^109^ More recently, randomized evidence has begun to clarify the relative benefits of proton therapy in this setting. The phase 3 Prostate Advanced Radiation Technologies Investigating Quality of Life (PARTIQoL) trial compared proton radiation to photon IMRT. The trial included 450 patients with low to intermediate risk prostate cancer. The primary end point was change in baseline bowel quality of life at 24 months. Secondary end points included changes in urinary and sexual function as well as treatment efficacy.^110^, ^111^ With a median follow‐up of 60.3 months, there was no difference between proton therapy and photon IMRT in bowel function at 24 months with excellent outcomes in both groups. Similarly, there was no difference in urinary function, sexual function, or PFS at 5 years.^24^ These findings suggest that despite advantage of a steeper dose fall off, proton therapy does not confer a measurable improvement in patient reported outcomes in an unselected population of patients. Future work is needed to identify patient subgroups most likely to benefit, potentially based on anatomy or baseline function.

Proton therapy may also offer advantages for patients requiring pelvic nodal irradiation in the definitive or postoperative setting. Compared with photon techniques, proton therapy can substantially reduce low and intermediate dose exposure to surrounding bowel, bladder, pelvic bone marrow, and other normal tissues in these settings.^112^, ^113^ Prospective clinical data support the safety and efficacy of proton therapy in these settings, including for pelvic node–positive prostate cancer requiring high doses of radiation therapy in the pelvis.^114^, ^115^, ^116^, ^117^

Beyond prostate cancer, proton therapy has also been explored in other genitourinary malignancies, including as a stereotactic ablative approach for primary renal cell carcinoma in medically inoperable patients, although clinical data in these settings remain limited.^118^

### Hematologic

The rationale for proton therapy among patients with hematologic malignancy is driven by the goal of minimizing long‐term toxicity in a population with high cure rates and prolonged survivorship. For patients requiring mediastinal or thoracic radiation, proton therapy reduces dose to nontarget structures such as the heart, lungs, and breast tissue compared with modern photon techniques.^25^, ^119^ The reduction in breast dose is particularly relevant for children and young adults receiving mediastinal radiation for lymphoma who are at a high lifetime risk of secondary breast cancer.^120^ The dosimetric advantages of proton therapy are estimated to meaningfully reduce lifetime risk of secondary breast cancer.^121^, ^122^ Furthermore, prospective phase 2^123^ and pooled multi‐institutional data^124^ have demonstrated the feasibility and efficacy of proton therapy among patients with mediastinal lymphoma, with low rates of acute toxicity and disease control outcomes comparable to historical photon based series.

### Reirradiation

Proton therapy can be particularly well suited for reirradiation scenarios where prior radiation exposure limits the tolerance of adjacent normal tissues and the steeper dose fall off of protons are most clinically meaningful. Evidence varies and includes retrospective and single arm prospective studies. For example, a multi‐institutional prospective registry study led by the Proton Collaborative Group evaluated proton radiotherapy for reirradiation among patients with recurrent glioblastoma (NCT01255748) showed that proton reirradiation was safe and feasible despite prior high dose intracranial irradiation.^26^ Similar, retrospective series have observed good safety and efficacy in recurrent breast cancer.^125^, ^126^ In head and neck cancer, both retrospective^26^, ^146^ and ongoing prospective efforts (NCT01973179 and NCT03981068) are informing the role of proton therapy in the setting of reirradiation including curative intent treatment.

### Future directions

Emerging technological advances may further expand the role of proton therapy in oncology and improve the therapeutic ratio for patients. Ultra‐high dose rate radiotherapy, termed FLASH radiation, has demonstrated the potential to reduce normal tissue toxicity while maintaining tumor control in preclinical studies. Proton radiation currently represents one of the most promising platforms for clinical translation of FLASH radiotherapy.^128^, ^129^, ^130^, ^131^ Early human clinical studies of proton FLASH radiation for patients with painful bone metastases have demonstrated feasibility and safety.^132^, ^133^ Advances in imaging and treatment planning have also facilitated the possibility of adaptive proton therapy, which allows for reoptimization of proton radiation planning during the course of treatment based intrafractional motion (such as breathing and organ motion) and interfractional motion (such as tumor shrinkage or weight loss).^134^, ^135^, ^136^ In addition, proton arc therapy is an emerging radiation delivery technique allowing for radiation from a large number of gantry angles or even continuous delivery rather than a small number of fixed beam angles, thereby improving conformality^137^, ^138^, ^139^ with encouraging results from the first in human studies among patients with head and neck cancer.^140^ Finally, technological advances in planning techniques, including RBE or LET informed planning that more accurately takes into account biological damage per unit of dose, may further reduce toxicity risk risks of proton therapy.^3^

Several advances may improve access to proton therapy. Although the number of proton facilities has increased rapidly, there remain only approximately 120 proton centers worldwide compared with more than 8000 photon radiotherapy facilities.^141^ Most proton centers are located in the United States, Europe, and Asia, creating geographic barriers to access for patients living in rural areas or low‐ and middle‐income countries.^141^, ^142^ One potential innovation to improve access is upright proton therapy, in which proton therapy is delivered while a patient is seated or standing rather than supine.^141^ By eliminating the need for a massive rotating proton gantry, upright proton therapy would significantly reduce the cost of new facility construction and may improve access to care.^143^

## CONCLUSION

Proton therapy has been in use for over 60 years with the expectation of target dose escalation and surrounding normal tissue sparing as the combined means to improve disease control and reduce treatment toxicity. Parallel advancements in photon technology have occurred so quickly that the margin of benefit of proton therapy is less clear. Nonetheless, with advancements in proton technology as well, it is expected that the physical advantage of the Bragg peak and finite beam length of proton beams will lead to true future clinical gains. Future directions in proton therapy will depend on demonstrating clinically meaningful benefit beyond dosimetric advantages. Ongoing and recently completed randomized trials across disease sites, including RADCOMP, RTOG 1308, and NRG GI006, are expected to provide critical data regarding the impact of proton therapy on survival, toxicity, and patient reported outcomes. Long‐term follow‐up will be essential, particularly in disease sites where late effects drive morbidity.

As these data mature, the role of proton therapy is likely to evolve toward a more selective application, focused on patient populations in whom reductions in normal tissue dose are most likely to translate into meaningful clinical benefit. A key question is how to identify which patients are most likely to derive meaningful clinical benefit from proton therapy. Although randomized trials remain the gold standard, patient selection will likely become increasingly individualized through integration of dosimetric, clinical, and biologic factors. Normal tissue complication probability modeling and model‐based selection may identify patients in whom the anticipated reduction in treatment‐related toxicity justifies proton therapy.^144^, ^145^, ^146^ In parallel, expected survival, competing risks of late toxicity, patient comorbidities, age, and baseline functional status should all inform treatment selection, as the greatest benefit from reduced integral dose may accrue to patients with longer life expectancy or heightened susceptibility to radiation‐induced complications. Advances in artificial intelligence may further refine patient selection by integrating dosimetric and clinical data to predict individualized benefit.^12^ ^,^ ^147^

## AUTHOR CONTRIBUTIONS

**Emily S. Lebow**: Conceptualization, writing–original draft, and writing–review and editing. **Helen A. Shih**: Conceptualization, supervision, writing–original draft, and writing–review and editing.

## CONFLICT OF INTEREST STATEMENT

Helen A. Shih reports the following: UpToDate, section editor and writer; AbbVie, research support to institution; Servier Pharmaceuticals, compassionate use panel, advisory board, educational program; Novocure, advisory board. The other author declares no conflicts of interest.
